# Cognitive tasks as measures of pig welfare: a systematic review

**DOI:** 10.3389/fvets.2023.1251070

**Published:** 2023-11-13

**Authors:** Thomas Ede, Thomas D. Parsons

**Affiliations:** Swine Teaching and Research Center, University of Pennsylvania School of Veterinary Medicine, Kennett Square, PA, United States

**Keywords:** cognition, affective state, cognitive bias, avoidance, animal welfare, swine, judgment bias, animal emotion

## Abstract

Cognitive approaches are increasingly used to assess animal welfare, but no systematic review has been conducted on pigs despite their cognitive capacities. Our aims were two-fold: first, to assess the popularity and heterogeneity of this approach by quantifying the different cognitive tasks used and welfare interventions studied. The second was to assess how often results from cognitive tasks supported treatment effects. The search yielded 36 studies that met our criteria. Eleven different cognitive tasks were applied (three most common: judgment bias, learned approach/aversion, and holeboard). Welfare interventions investigated were also diverse: the impact of 19 other different events/conditions/states were reported (most common: housing enrichment). We defined “supportive” as the observation of a significant difference between treatment groups consistent with an author’s expectation or hypothesis. Supportive findings were reported in 44% of papers. Interventions yielded no significant difference in 33% of studies. In another 21% of reports, outcomes were mixed and a single study refuted the author’s predictions. When considering specific cognitive tasks, authors’ predictions of welfare differences were supported most often when using learned approach/aversion (55% of these studies). Similar supportive results were observed less commonly (40% each) when using judgment bias and holeboard tests. Analysis of additional concomitant measures of welfare (health, physiology or behavior) revealed that behavioral measures were most frequently supportive of author’s expectations (41%) as well as often matching the actual outcomes of these cognitive tasks (47%). This systematic review highlights the growing popularity of cognitive tasks as measures of pig welfare. However, overall rates of supportive results, i.e., changes in performance on cognitive tasks due to welfare interventions, have been limited so far, even for the most employed task, judgment bias. The numerous different combinations of experimental paradigms and welfare interventions reported in the literature creates challenges for a critical meta-analysis of the field especially in evaluating the efficiency of specific cognitive tasks in assessing animal welfare. This work also highlights important knowledge gaps in the use of cognitive tasks that will require both further validation as well as novel innovation to ensure that their potential is fully realized in the measurement of pig welfare.

## Introduction

Animal welfare is a growing topic in societal discourse, with concerns over the way farm animals are raised (1). Efforts have been made to define animal welfare, but no clear consensus has been reached. One prominent view divides welfare in three overlapping components (2): health, natural living, and affective states. Other definitions have been introduced (3–5) but authors generally agree on the importance of animals’ affective or emotional states, some even arguing this component to solely be relevant (6, 7). Unfortunately, the subjective nature of emotions creates a challenge in the objective measure of animal welfare. An animal’s subjective experiences are still considered outside the reach of direct scientific inquiry (8) but proxy measures based on health, behavior, physiology and – more recently – cognitive approaches have been developed (9, 10).

Studies that recognize the importance of animals’ cognitive and emotional states have gained considerable popularity over recent years because of the interdependence between emotions and cognitive processes. Emotional states can impact cognition whereas cognition influences emotions and hence welfare (9, 11, 12). Emotions are commonly defined by two components: valence (positive or negative) and arousal (i.e., level of activation) (10). Emotional valence is integral to animal welfare which entails minimizing negative experiences and maximizing positive ones. As cognitive approaches are especially relevant to the study of emotional valence (9), their application to animal welfare studies has gained popularity in recent years. Despite being still novel in the field of animal welfare, the use of cognitive measures has been applied to many species of farm animals. For example, dairy cattle exhibit a negative judgment bias (i.e., a more pessimistic outlook when presented with an ambiguous stimulus) after a painful procedure (13) or separation from the dam (14), and sheep exhibit a positive bias from released from restraint (15). Cognitive approaches also bolster the assessment of positive welfare states (11). Whereas historically popular health parameters such as mortality or productivity often identified only negative states of welfare (16), cognitive paradigms have gained traction in the study of positive welfare. For example, pigs exhibit a positive bias (i.e., more optimistic outlook) when housed in an enriched environment (17).

Several reviews have been published on the use of cognitive tasks to assess animal welfare in various species (18–24), reflecting the growing interest in this approach. However, no systematic review has been conducted on pigs despite their complex cognitive capacities such as tool-use (25), deception (26) or playing a video-game (27). Given their advanced cognitive capacity, pigs have a great potential to suffer, and concerns have been raised about common practices in pig rearing such as barren environments (28), restriction of movement (29), and painful procedures (30–32). Thus, cognitive approaches represent an important opportunity for assessing pig welfare.

This review provides a comprehensive and systematic overview of the current knowledge on cognitive measures of pig welfare. The field is still relatively new and employs a variety of methods. Thus, it would be premature to provide a quantitative, statistically valid meta-analysis of this literature. First, we aim to assess the popularity and heterogeneity of this approach in the literature. A descriptive section details the various experimental paradigms found in our search including: cognitive tasks used, welfare interventions studied, their expected effects on welfare as stated by authors (i.e., whether the intervention was hypothesized to have a positive or negative impact on welfare), as well as additional measures of welfare (e.g., health, physiological, and behavioral parameters). Second, we aim to assess how often results of these studies support authors’ hypotheses. We will link the previous parameters (tasks, interventions and hypothesized effects) to the outcomes of each study (i.e., whether the authors’ hypothesis was supported). Finally, we intend to gain insight into whether specific combinations of cognitive tasks and interventions have led to supportive outcomes, and to determine the agreement between cognitive tasks and other measures of welfare.

## Methods

The PICOS (Population, Intervention, Comparison, Outcome, Study type) framework (Table 1) was used to formulate our search (33). We conducted the systematic review in April 2023 on the Web of Science database with no limit on date, with the following search terms:

**Table 1:** Specifications of the Population, Intervention, Comparison, Outcome, Study type (PICOS) framework components used for the systematic review.

Population/Participants	Swine of any age, breed or sex
Intervention/exposure	Events/conditions/states potentially affecting welfare. Examples: Farm practices: housing type, handling technique, social mixing, isolation… Physiological or physical factors: life or gestation phase, birthweight, deficiencies, supplements…
Comparison	Inter-subject designs (treatment groups compared to a control) Intra-subject designs (comparison to subject’s own baseline, crossover treatments)
Outcomes	Primary:**cognitive task**, intervention,**hypothesized valence of intervention**,**outcome**. *See below for definitions* Secondary: Authors, publication year, sample size, number of treatment groups, pig breed, sex and age, additional welfare measures
Study type	Peer-reviewed experimental articles (no gray, reviews or conference papers) written in English. Studies need to both: Include a cognitive task Apply this task as a welfare measure

[All fields]: (cognit* or learn*) and (welfare or affective or emotion*).

And [Title]: (pig* or sow* or gilt* or boar* or swine or sus scrofa)

*Not [Title]: guinea.*

### Definitions

#### Cognitive task

These experimental paradigms, as adapted from Kester and Kirschner (34) require “*a subject to mentally process new information (*i.e.*, acquire and organize knowledge/learn) and allow them to recall, retrieve that information from memory and to use that information at a later time in the same or similar situation (i.e., transfer).*” Examples of this approach include judgment biases, mazes, learned approach/aversion, and puzzle boxes. This review did not consider studies making measurements in the absence of a learning process, such as reflex behaviors, physiological responses, health measures, or personality tests (e.g., open field, novel object, startle tests). For a detailed discussion on these measures, see (35). Furthermore, studies that only included cognitive tasks as a dependent variable (e.g., as an enrichment treatment) were not considered. Finally, we did not include studies exploring cognition in a basic/fundamental or methodological perspective (e.g., social learning, use of mirror, joystick, call-feeding-station) without a direct application to welfare assessment.

#### Hypothesized valence of intervention

The different welfare interventions were grouped by their *a priori* hypothesized positive or negative impact on animal welfare. We relied on the authors’ stated predictions or expectations. For example, housing enrichment was expected to increase welfare, so it was classified as a hypothesized *positive* intervention. Stunning gases were expected to compromise welfare, hence was classified as a hypothesized *negative* intervention. When authors had a hypothesized general treatment effect, without a specific direction stated, the hypothesized valence was reported as *Indeterminant.*

*Outcomes* were rated in relation to each study’s hypothesis. Results were categorized among four possibilities:

- *Supportive*: clear significant differences in cognitive task outcomes between treatment groups consistent with an author’s expectation or hypothesis about the welfare intervention. For studies with an *Indeterminant* hypothesized valence, any treatment effect (regardless of direction) was reported as supportive of the hypothesis.
- *Not supportive:* no significant cognitive task outcomes between treatment groups dictated by the welfare intervention
- *Mixed*: some but not all the outcomes between treatment groups have a significant effect or tendency, often the result of a post-hoc subset of measures or population
- *Refuted:* clear significant differences in cognitive task outcomes between treatment groups that contradict an author’s expectation or hypothesis about the welfare intervention

These definitions of outcomes cannot solely be used to evaluate the suitability of the cognitive task to detect welfare differences, and should be considered in combination with the intervention studied. The lack of significant outcome arises from either the inability of the behavioral assay to adequately assess welfare states or that there may be no difference between the welfare of the treatment groups. The latter could result from the chosen experimental intervention not being sufficient to elicit a welfare change rather than a failure in the cognitive task to detect a change. We encourage readers to keep in mind the inextricable relationship between sensitivity of cognitive task and effect size of intervention throughout our manuscript.

Additional measures of welfare were extracted, as well as their outcomes (*Supportive, Not supportive, Mixed*, see previous definition). Considering the heterogeneity of these measures, they were grouped in four categories:

- Health: growth, lesions, lameness, Body Condition Score, inflammation
- Physiology: cortisol (serum, saliva, hair), alpha-amylase, dopamine, serotonin, microRNA
- Behavior: posture, activity budgets, aggression, vocalizations, retreat/escapes, gasps, Qualitative Behavioral Assessment, Novel Environment, Novel Object, Novel human tests

Details on which specific measure(s) were reported in each study and their outcome is detailed in Supplementary material 1. Pooled categories are presented in Table 2.

**Table 2:** Studies fitting the PICOS framework detailed in Table 1.

Cognitive task	Intervention/Exposure	Hypothesized Valence of intervention	Outcome Cognitive task	Health	Behavior	Physiology	Age	Sample size	Breed	Sex	Year	Authors
							**(weeks)**	**(# groups)**				
Judgment bias	Serotonin depletion	Negative	Supportive		Not supportive		8	48 (2)	German Landrace	F	2017	Stracke et al.
												(36)
Judgment bias	Housing enrichment	Positive	Supportive				12	10 (2)	Large White × Landrace	F	2012	Douglas et al.
												(17)
Judgment bias	Handling/Human contact	Positive	Supportive				4	54 (3)	(Yorkshire × Landrace) × Duroc	Mx	2015	Brajon et al.*
												(37)
Judgment bias	Gestation	Negative	Supportive				34	20 (1)	Large White, Landrace, Duroc	F	2022	Bushby et al.
												(38)
Judgment bias	Hierarchy	Indeterminant	Supportive				Mixed parity	24 (1)	PIC 1050 Landrace-Yorkshire	F	2019	Horback and Parsons
												(39)
Judgment bias	Rearing stage – Personality	Indeterminant	Supportive	Not supportive			55	25 (1)	PIC 1050, Landrace-Yorkshire	F	2022	Horback and Parsons
												(40)
Judgment bias	Isolation/Restraint	Negative	Not supportive				7	32 (2)	German Landrace	F	2013	Dupjan et al.
												(41)
Judgment bias	Isolation/Restraint	Negative	Not supportive				22	15 (2)	Göttingen minipigs	F	2013	Murphy et al.
									Duroc × Yorkshire			(42)
									Duroc × Danish Landrace			
Judgment bias	Stocking density	Negative	Not supportive	Mixed	Mixed	Not supportive	14	40 (2)	Large White × Landrace	Mx	2014	Scollo et al.
												(43)
Judgment bias	Handling/Human contact	Negative	Not supportive		Not supportive	Not supportive	23	56 (2)	(Landrace × Large White) × Piétrain	F	2017	Carreras et al.
												(44)
Judgment bias	Handling/Human contact	Negative	Not supportive				4	54 (3)	(Yorkshire × Landrace) × Duroc	Mx	2015	Brajon et al.*
												(37)
Judgment bias	Housing enrichment	Positive	Not supportive	Supportive	Supportive	Supportive	16	44 (2)	Large White × Landrace	F	2016	Carreras et al.
									with Piétrain heterozygous			(45)
Judgment bias	Housing enrichment	Positive	Not supportive			Mixed	5	24 (2)	Large White × Landrace	F	2021	Marsh et al.
												(46)
Judgment bias	Low birthweight	Negative	Not supportive			Not supportive	5	42 (2)	(Yorkshire × Dutch Landrace) × Duroc	Mx	2019	Roelofs et al.
												(47)
Judgment bias	Housing enrichment	Positive	Mixed		Supportive		7	36 (2)	Large White × Landrace	Mx	2016	Asher et al.
												(48)
Judgment bias	Serotonin supplement	Positive	Refuted		Not supportive		8	32 (2)	German Landrace	F	2017	Stracke et al.
												(49)
Learned approach/aversion	Stunning gas	Negative	Supportive		Supportive		22	Exp 1: 12 (1)	Halothane-free	F	2010	Dalmau et al.
								Exp 2: 12 (1)				(50)
Learned approach/aversion	Injection methods	Negative	Supportive	Supportive	Supportive	Not supportive	4	36 (3)	(Landrace × Large White) × Pietrain	Mx	2021	Dalmau et al.
												(51)
Learned approach/aversion	Electric prodder	Negative	Supportive				17		Large White × Landrace	M	2000	Jongman et al.*
								Exp 2: 30 (3)				(52)
Learned approach/aversion	Isolation/Restraint	Negative	Supportive		Supportive		35	12 (1)	NS	F	1998	Špinka et al.
												(53)
Learned approach/aversion	Stunning gas	Negative	Supportive		Supportive		22	Exp 1: 16 (1)	(Duroc × Landrace × Large White) x	F	2007	Velarde et al.
								Exp 2: 16 (1)	(Pietrain × Large White)			(54)
Learned approach/aversion	Handling/Human contact	Positive	Supportive	Not supportive			20	24 (3)	Large White × Landrace	F	1996	Hemsworth et al.*
												(55)
Learned approach/aversion	Stunning gas	Negative	Not supportive		Supportive		22	60 (3)	NS	F	2012	Llonch et al.
												(56)
Learned approach/aversion	Handling/Human contact	Negative	Not supportive	Not supportive			20	36 (3)	Large White × Landrace	F	1996	Hemsworth et al.*
												(55)
Learned approach/aversion	Handling/Human contact	Positive	Not supportive		Mixed		7	54 (3)	(Yorkshire × Landrace) × Duroc	Mx	2016	Brajon et al.*
												(57)
Learned approach/aversion	Stunning gas	Negative	Mixed				17	Exp 1: 30 (3)	Large White × Landrace	M	2000	Jongman et al.*
								Exp 3: 28 (2)				(52)
Learned approach/aversion	Handling/Human contact	Negative	Mixed		Mixed		7	54 (3)	(Yorkshire × Landrace) × Duroc	Mx	2016	Brajon et al.*
												(57)
Holeboard	Housing enrichment	Positive	Supportive	Supportive		Not supportive	11	20 (2)	Duroc × (Terra × Finnish Landrace)	F	2016	Grimberg-Henrici et al.
												(58)
Holeboard	Low birthweight	Negative	Supportive	Supportive		Mixed	8	40 (2)	(Yorkshire × Dutch Landrace) × Duroc	Mx	2018	Roelofs et al.
												(59)
Holeboard	Iron deficiency	Negative	Not supportive	Not supportive			10	20 (2)	(Terra × Finnish landrace) × Duroc	Mx	2016	Antonides et al.
												(60)
Holeboard	Mixing stress	Negative	Not supportive				17	20 (2)	Finish Landrace × York F1	F	2009	Arts et al.
												(61)
Holeboard	Large litter	Negative	Mixed	Mixed	Not supportive		12	20 (2)	T40 × Pietrain, Large White × 426 PIC	M	2016	Fijn et al.
												(62)
Maze	Housing enrichment	Positive	Mixed			Supportive	15	48 (2)	Great Yorkshire ×	Mx	2000	de Jong et al.
									(Great Yorkshire × Dutch Landrace)			(63)
Maze	Early socialisation	Positive	Mixed		Not supportive		8	100 (2)	(Large White × Landrace) × Duroc	Mx	2020	Weller et al.
Puzzle box												(64)
Operant task	Housing enrichment	Positive	Supportive				16	84 (2)	Large White × Landrace	Mx	2000	Sneddon et al.
Maze												(65)
Pig gambling task	Housing enrichment	Positive	Supportive	Not supportive		Mixed	15	20 (2)	Duroc × Yorkshire and Duroc	M	2017	van der Staay et al.
									x Danish Landrace			(66)
Pig gambling task	Low birthweight	Negative	Supportive	Supportive		Not supportive	10	16 (2)	Duroc × Yorkshire and Duroc × Danish Landrace	M	2015	Murphy et al.
Judgment bias												(67)
T Maze	Housing enrichment	Positive	Mixed	Mixed	Mixed	Not supportive	9	96 (4)	Large White × Landrace	Mx	2018	Ralph et al.
Executive function												(68)
T Maze	Growth retardation	Negative	Mixed				5	42 (4×4)	Large White × Duroc	Mx	2019	Schmitt et al.
Spontaneous Object Recognition								47 (4×3)				(69)
Water maze	Cognitive task	Positive	Mixed	Not supportive	Mixed	Not supportive	2	27 (3)	NS	Mx	2008	Siegford et al.
												(70)

Ages were converted to weeks for a uniform measure, when an age range was presented, an approximate median was reported. Asterisks (*) denote duplicated studies in the table due do the investigation of multiple welfare interventions. NS: Not specified, M: Male, F: Female, Mx: Mixed.

**Figure 1:**
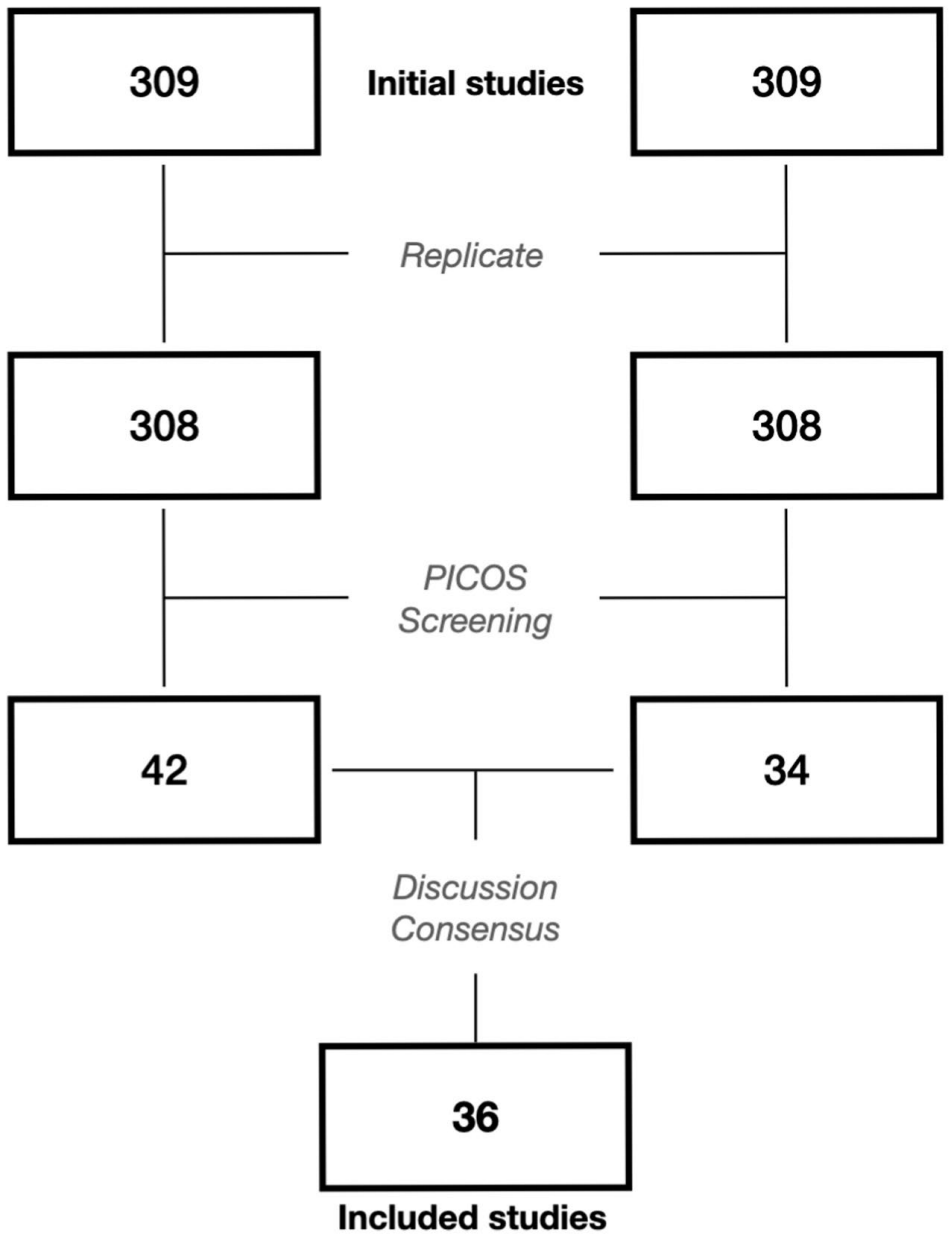
Exclusion–Inclusion flowchart for both raters. Studies from the initial disagreement between raters can be found in Supplementary material.

Two raters independently applied the PICOS framework previously outlined. Agreement was measured for the initial search and sources of disagreement were discussed before reaching a consensus on studies to include in the review. The Web of Science database yielded 309 articles (Figure 1), one replicate was found, and initial inclusion agreement between the two raters was 93.4%. Disagreements mostly stemmed from the ambiguous line between fundamental and applied studies and the explicit use of cognitive tasks as a welfare measure. After a discussion between the two raters, a consensus was reached to include the 36 studies detailed in Table 2. The list of studies excluded by discussion can be found in Supplementary material 2.

## Results

Findings are presented in two sections.^1^ The first is a descriptive section that details the distribution of general information from the studies (year, breed, age, sex, sample sizes), cognitive tasks used, and welfare interventions studied, as well as their grouping by hypothesized valences. Whereas the second section focuses on the study outcome (i.e., whether the hypothesis was supported, not supported, mixed findings or refuted) in relation to the cognitive task used, the welfare intervention and the hypothesized valence of the intervention. We also detail study outcomes for the most common cognitive task and intervention combinations.

## Section 1: descriptive results

### General information

The year of publication ranged from 1996 to 2022. The growing popularity of cognitive approaches to welfare assessment is reflected in our results, with the majority of studies published in the last 10 years. Various breeds were used across studies, with the most common being Landrace (n = 30), Large White (16), Duroc (13), and Yorkshire (11). The age of subjects ranged from 2 weeks to multiparous. Interestingly, no pigs under 2 weeks old have been studied likely due to difficulty the training young pigs to cognitive tasks. The average age of animals in this review was still relatively young at 14.5 ± 10.7 (SD) weeks (mixed parity study excluded). Few studies only included males (11%), while the distribution between females only (50%) and mixed (39%) was more balanced. On average, 17 ± 10 (SD) animals were enrolled per treatment group. This is a relatively low but not unexpected sample size due to the high workload and time commitment associated with the cognitive task experimental paradigms. Notably, Weller et al. (64) and Sneddon et al. (65) had the highest number of animals per treatment group, 50 and 42, respectively.

### Cognitive tasks

Various cognitive tasks were found, with 11 paradigms applied in the literature. However, their usage was not similar, with a few tasks applied more often than others. The three most common paradigms were:

1. judgment biases (17 studies): a task where animals are trained to discriminate between positive (e.g., food reward) and negative (e.g., air puff, absence of food) stimulus based on specific cues (e.g., location, color, auditory). Subjects are then presented an ambiguous cue, and their response is considered a proxy for their emotional state: If they react as if the ambiguous cue indicates positive stimuli, they are considered positively biased or “optimistic” (i.e., in a positive affective state); if they react as if the cue is negative, they are negatively biased or ‘pessimistic’ (i.e., in a negative affective state) (9, 71). For example, gilts currently housed in an enriched environment were faster to approach an ambiguous auditory cue, suggesting more positive welfare (17).
2. The second most common paradigm was learned approach/aversion (11 studies), where animals learn to associate cues (e.g., a specific environment) with stimuli (e.g., interaction with a handler). If animals are eager to return to that place (i.e., low latency to return, high time spent in that environment) even in the absence of the stimulus, that stimulus is deemed to have induced a positive experience. On the other hand, if the animals’ reaction is avoidance, the stimulus is assumed to have caused a negative experience (72). For example, pigs were more reluctant to re-enter an environment where they had previously been exposed to carbon dioxide compared to atmospheric air. This aversion was more marked with higher CO2 concentrations (54), suggesting that CO2 induces a negative association between affective experience and environment.
3. Finally, the holeboard test (5 studies) is a spatial discrimination task allowing the assessment of cognitive performances and behavioral flexibility by presenting subjects with holes (commonly 16), some baited with food rewards, some empty. For example, when compared to pigs housed in a barren environment, enriched pigs had better performances in the task (i.e., faster search, reduced visits to unbaited holes or holes already visited). The remaining paradigms, such as pig gambling task (66), were not as common with 3 or less studies.

### Welfare interventions and hypothesized valence

The welfare interventions investigated were diverse, with the impact of 19 different events/conditions/states studied across the literature. Housing enrichment was the most popular type of intervention done, with 11 investigations [e.g., (33, 35)]. Other common interventions were handling techniques [*n* = 7, e.g., (62)] and stunning gases [*n* = 4, e.g., (51)].

When grouping studies by expected intervention valence, the distribution was relatively well balanced, with 22 studies of negative valences [e.g., stunning gas (54), isolation/restraint (53)] and 15 positives [e.g., housing enrichment (46)]. Two studies did not have clear predictions on the directionality of the welfare interventions studied [personality trait (40) and feed rank (39)].

## Section 2: outcome

### Cognitive tasks

**Figure 2:**
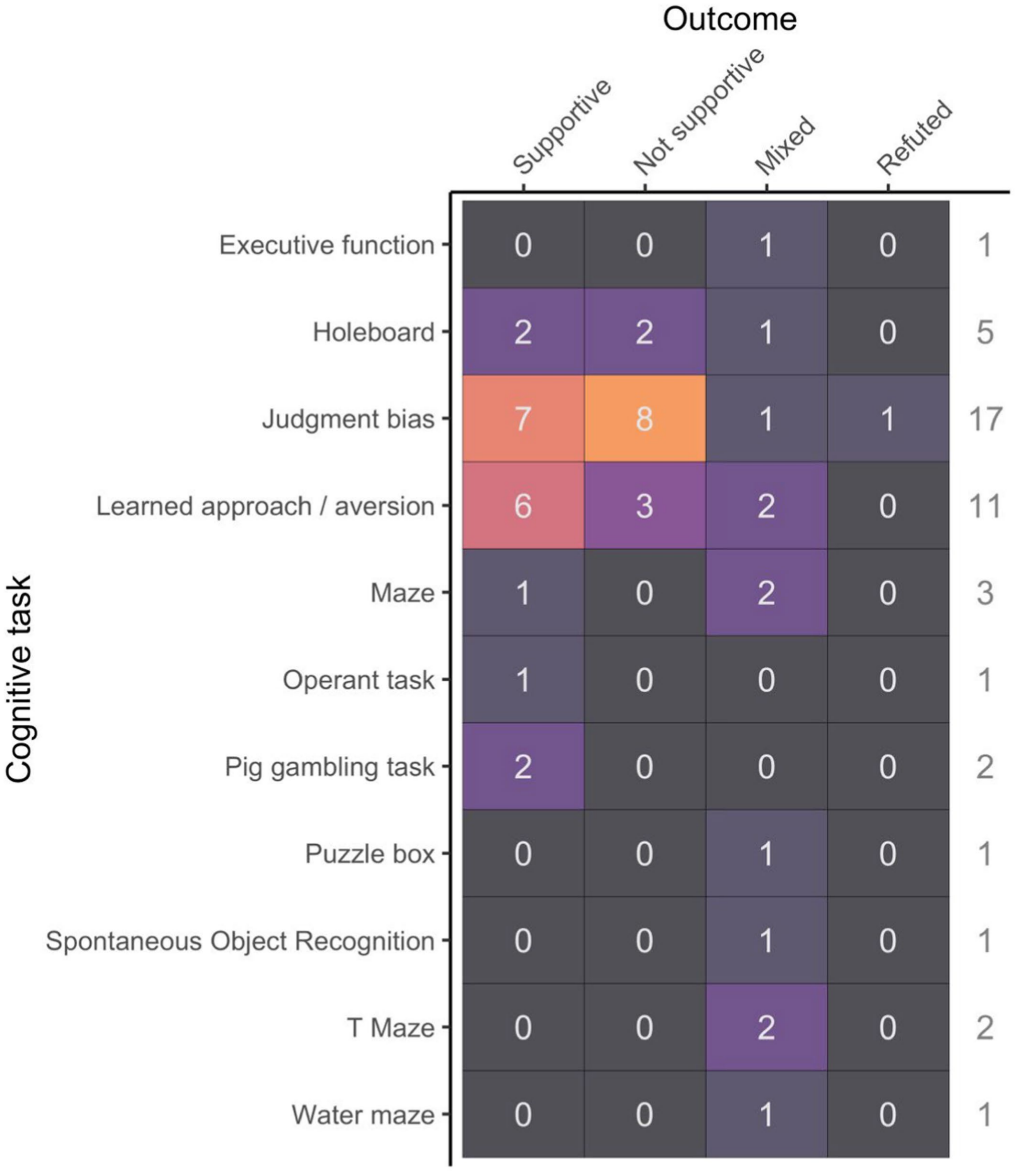
Study outcome (supportive, not supportive, mixed or refuted) in relation to the cognitive task used.

Overall, 44% of studies yielded supportive outcomes (i.e., the experimental intervention translated to an effect on the cognitive task consistent with authors’ hypothesis). 33% of studies yielded unsupportive results (i.e., no treatment effect was found), and 21% yielded mixed outcomes.^2^ A single study refuted the authors’ hypothesis (49). Among the most popular paradigms, rates of support of authors’ hypothesis varied, with a maximum of 55% for learned approach/aversion experiments, whereas supportive results were observed only about 40% for judgment bias and holeboard tests (Figure 2). No correlation was found between the average sample size per treatment group and rates of supportive results for the three most common cognitive tasks (*t* = −0.3, *p* = 0.8).

### Welfare interventions and hypothesized valence

**Figure 3:**
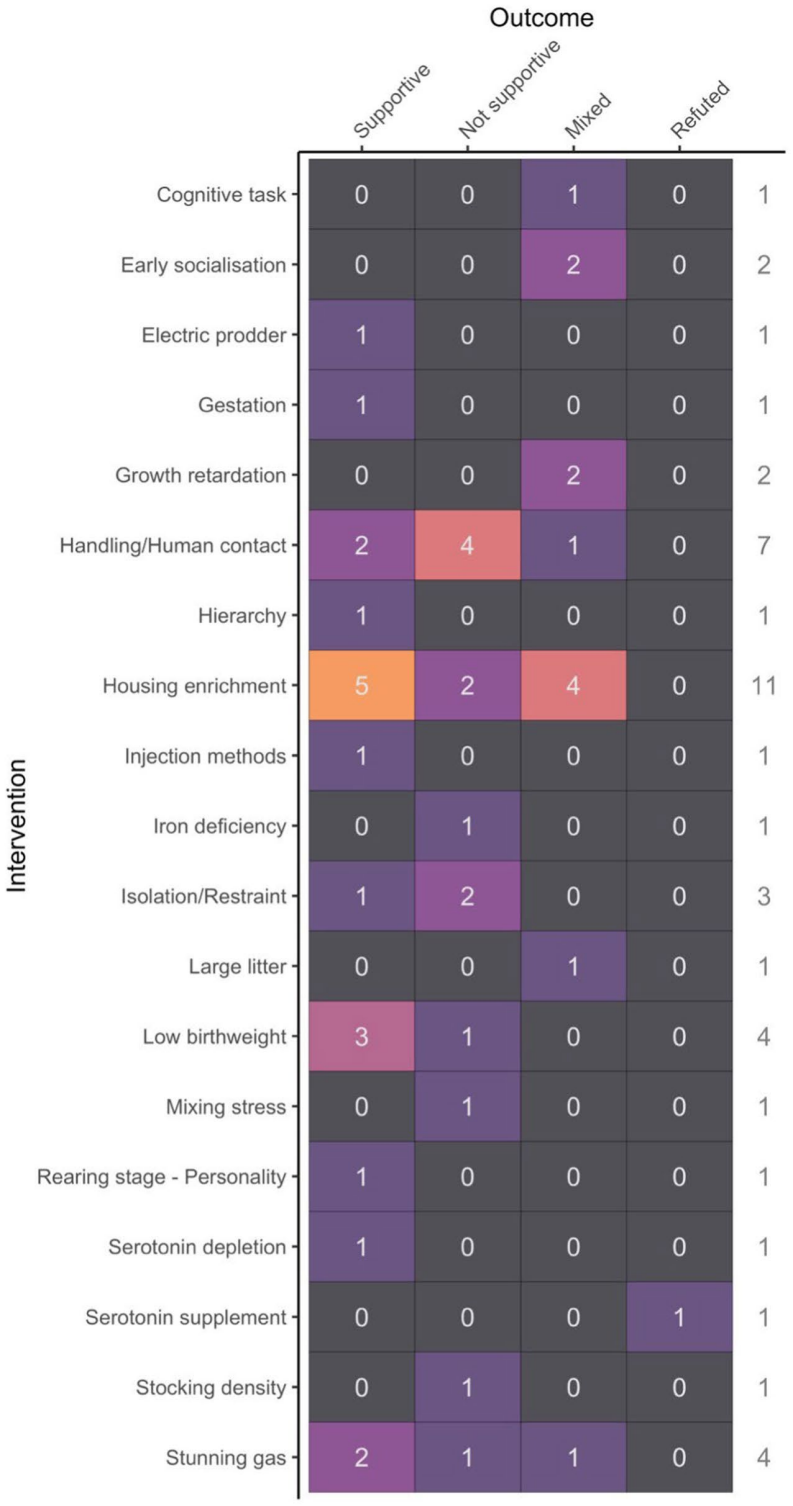
Study outcome (supportive, not supportive, mixed or refuted) in relation to the intervention studied.

Only five specific welfare interventions were assessed in at least three studies, and rates of studies with supportive results varied from only 29% for handling/human contact to 75% for birth weight. In-between levels of supportive results included: isolation/restraint at 33%, housing enrichment at 45%, and stunning gas at 50% (Figure 3). When considering these five most common interventions, no correlation was found between the average sample size per treatment group and rates of supportive results (*t* = −0.3, *p* = 0.8).

**Figure 4:**
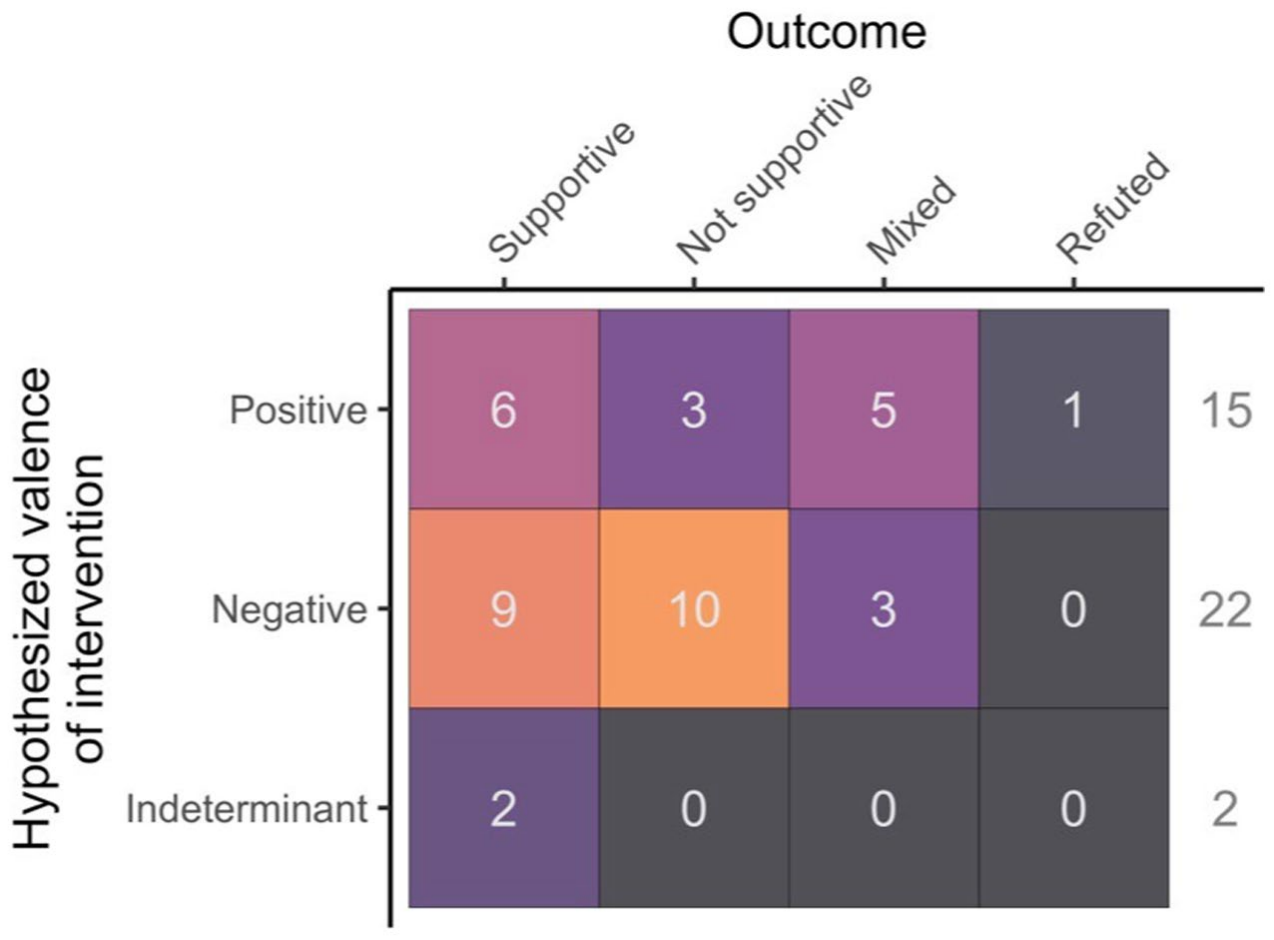
Study outcome (supportive, not supportive, mixed or refuted) in relation to the hypothesized valence of the intervention (positive, negative or indeterminant).

When grouping interventions by hypothesized valence, rates of supportive results were similar for both positive and negative valences at 40 and 41%, respectively. Both studies with an indeterminant hypothesized valence reported a significant effect of the factor studied, which included personality trait (40) and feed rank (39) (Figure 4).

### Cognitive task × intervention combinations

**Figure 5:**
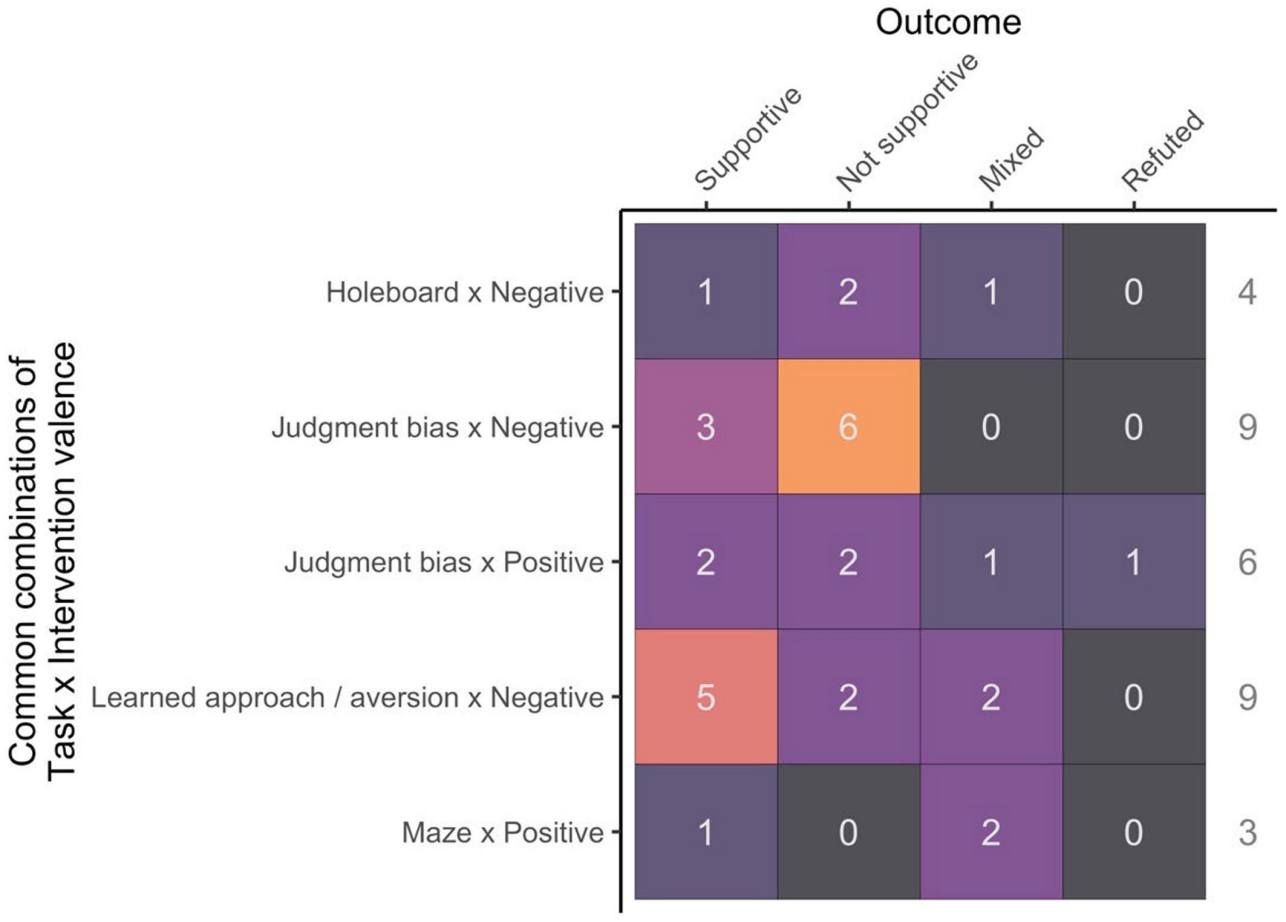
Study outcome (supportive, not supportive, mixed or refuted) in relation to the combination between cognitive task used and hypothesized valence of the intervention (positive, negative or indeterminant). The most common combinations (minimum of 3 studies) are shown.

Five combinations of cognitive task and hypothesized valence of intervention were found in at least three different studies. This included: judgment bias with a negative intervention (*n* = 9), learned approach/aversion with a negative intervention (*n* = 9), judgment bias with a positive intervention (*n* = 6), holeboard with a negative intervention (*n* = 4), and maze with a positive intervention (*n* = 3). The combination of a negative intervention with a learned approach/aversion yielded the most supportive results of the author’s expectations (55% of the time). On the other hand, a judgment bias outcome supporting the author’s expectation was observed in 33% of cases for either positive or negative expected valence interventions. Interestingly the only study where the author’s expectations were refuted was a judgment bias task in combination with a positive intervention (49). The two remaining combinations, holeboard with a negative intervention and maze with a positive intervention both had low rates of supportive results at 25 and 33%, respectively (Figure 5). No correlation was found between average sample size per treatment group and rate of supportive results (*t* = −0.2, *p* = 0.8).

### Additional measures

**Figure 6:**
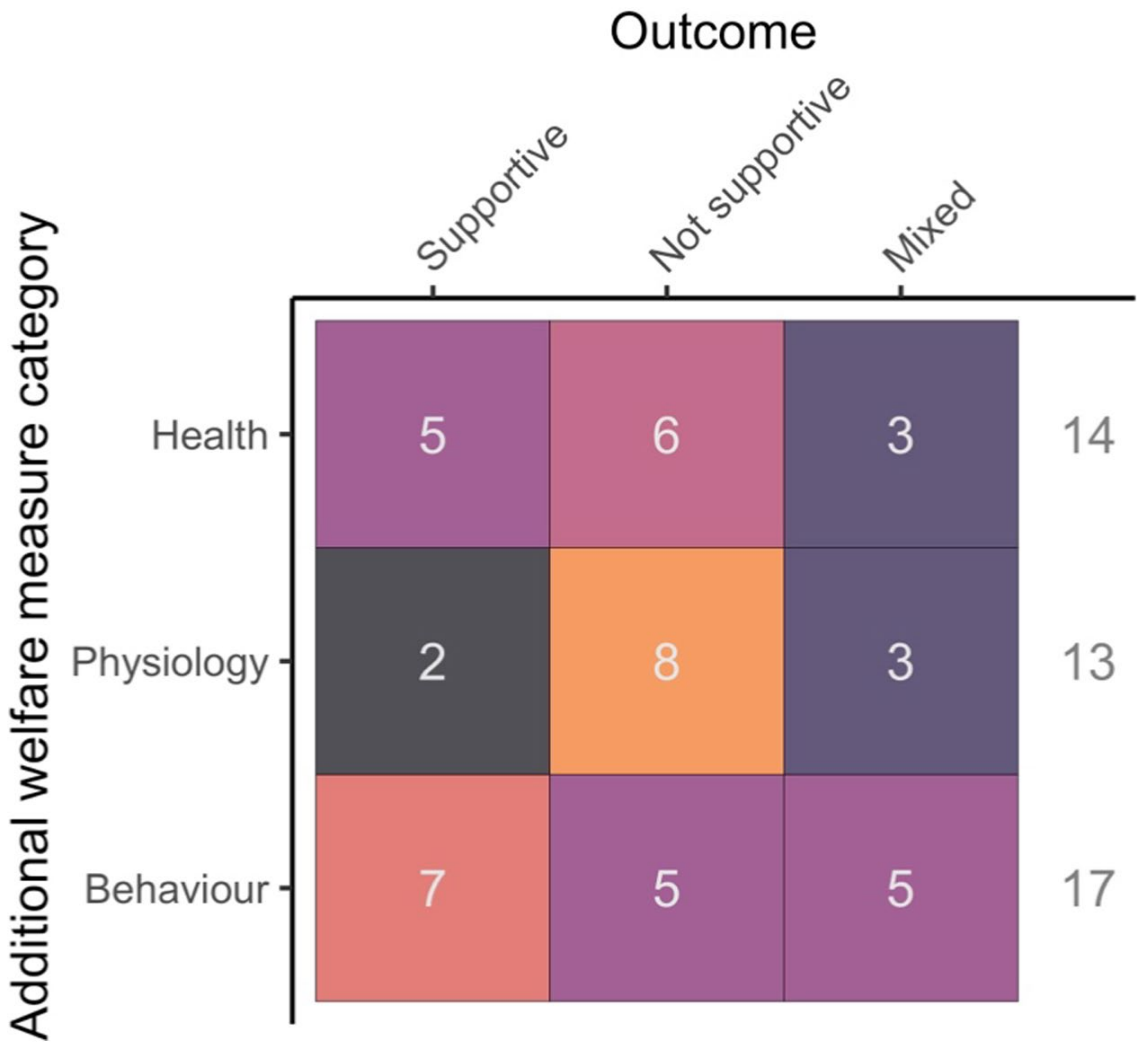
Outcome of additional welfare measure (hypothesis supported, not supported or mixed) in relation to the category of the measure (Health, Physiology or Behavior).

Health parameters were reported in 14 studies, physiological measures in 13 and behavioral observations in 17 while 12 studies did not use additional measures of welfare. There were great discrepancies in rates of supportive results (i.e., a reported treatment effect in alignment with expectations), with behavior the highest (41% of studies), whereas the other categories were lower (health: 36%, physiology: 15%, see Figure 6 for details).

**Figure 7:**
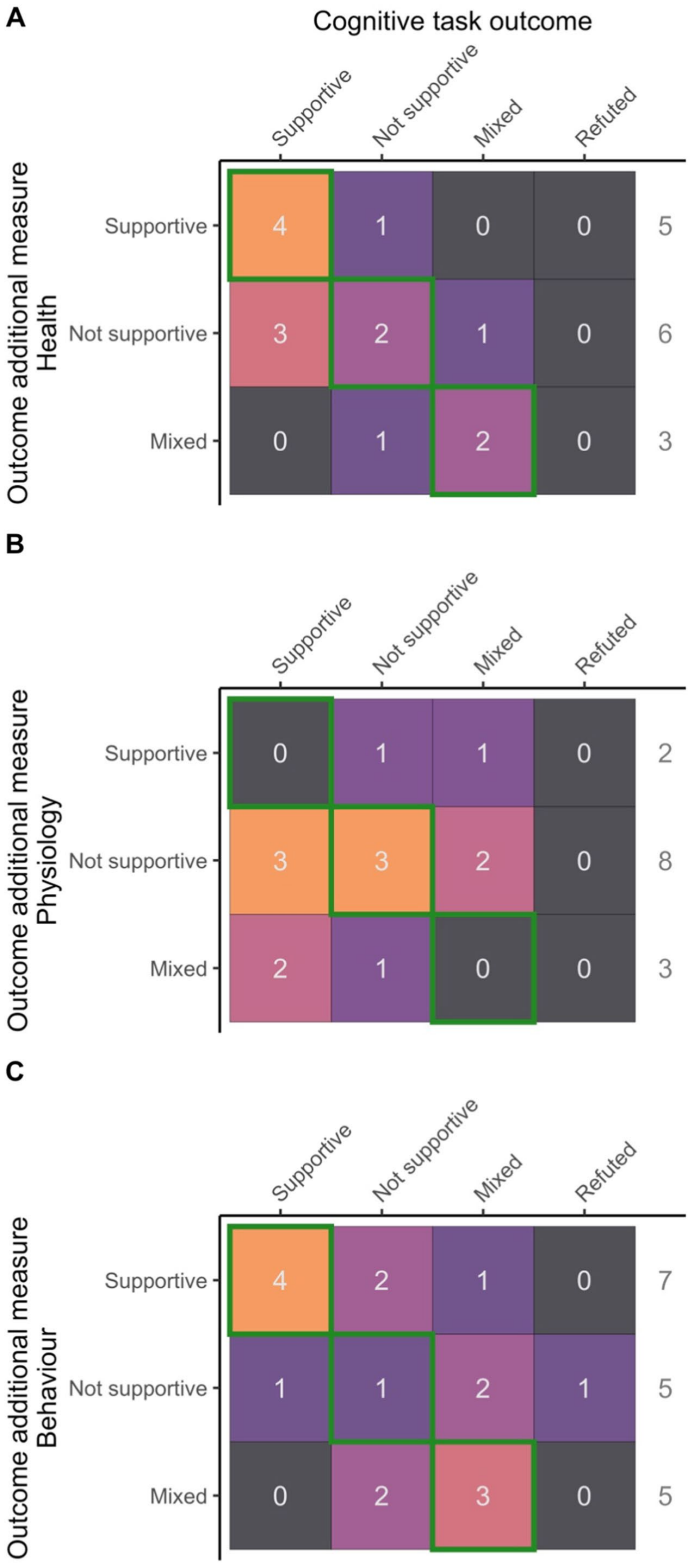
Consistency between outcomes (hypothesis supported, not supported or mixed) from cognitive tasks and additional measures of welfare [**(A)**: Health, **(B)**: Physiology, **(C)**: Behavior]. Cells contoured in green reflect matching outcomes.

When looking at the outcome consistency with cognitive tasks (i.e., whether both additional welfare measures and cognitive tasks had supportive, not supportive or mixed results, see Figure 7 for details), health and behavior had the highest consistency with cognitive tasks (57 and 47%, respectively, of studies reporting matching results) whereas physiology was lower (23%). There were 8 instances where outcomes of both additional welfare measures and cognitive tasks matched, as well as supported the authors hypothesized outcome: 4 from health measures (21, 58, 59, 67) and 4 from behavioral observations (50, 51, 53, 54). Interestingly, all four of the studies with matching supportive results from cognitive and behavioral approaches had adopted a learned approach/aversion paradigm to study negative interventions. Out of the 44 uses of additional welfare measures, there were only 4 instances where additional measures were supportive of authors’ hypothesis while the cognitive task outcome did not (45, 56), 3 of which were in the same study (45).

### General discussion

Our review of the literature shows that cognitive tasks as a measure of swine welfare is still an evolving and heterogeneous field but has been gaining traction in recent years. Various cognitive paradigms (e.g., judgment bias, learned approach/aversion) have been applied to assess the welfare impact of many different interventions (e.g., housing enrichment, stunning gases). The main finding of our work is the heterogeneity of the literature, as it was rare to find multiple studies using the same cognitive task to measure the same or similar welfare interventions. Unfortunately, this heterogeneity is expected due to researchers, funding agencies and publishers’ higher interest for original work over replication studies, as well as ethics committees’ reticence to approve previously conducted research. The relative infancy of the field also entails a lack of methodological standardization.

Judgment biases and learned approach/aversion were the most used paradigms among the 11 cognitive tasks identified, but several other less common yet creative methods have been employed. For example, Weller et al. (64) tested pigs’ innovation (i.e., ability to solve a new problem or find a new solution to an existing issue) by exposing them to a puzzle box they had to resolve to access a reward. Pigs’ betting tendencies were also studied via the Pig Gambling Task, with barren-housed or low birthweight pigs preferring “low-risk, low-reward” over “high-risk, high-reward” gambles (66, 67). However, given the limited number of studies exploring these novel methods, their suitability for measuring pig welfare requires further research.

We note that the different cognitive tasks used did not necessarily assess the same processes. For example, judgment biases and learned approach/aversion were applying a cognitive approach to test the affective impact of interventions, whereas tests like the holeboard or mazes were assessing the effect of interventions on cognitive abilities. Because of this different focus on either emotional or cognitive processing, we do not expect all cognitive tasks to be uniformly impacted by welfare interventions.

Welfare interventions were varied yet skewed in ways similar to cognitive tasks. Among the 19 different interventions, housing enrichment was the most common experimental manipulation. Handling/human contact, stunning gases, low birthweight, and isolation/restraint were less researched but still studied several times. Other interventions, such as injection methods (51), litter size (62), or serotonin levels (49), were only considered in single studies. When grouping the interventions by their hypothesized impact on welfare, we found studies that examined interventions expected to have a negative impact on welfare were slightly more common. This is consistent with the prevalence of conditions and procedures likely to induce negative welfare states in farm animals. However, a substantial number of newer studies are making use of cognitive tasks to explore positive welfare states. These studies are consistent with a growing appeal for Positive Animal Welfare in the last decades (11, 73, 74) and motivate scientists to seek novel animal welfare metrics measuring the impact of positive interventions.

Our results also highlight the overall relatively low rate of supportive results, i.e., changes in performance on cognitive tasks due to welfare intervention. Approximately 40% of the studies supported the authors’ hypotheses or expectations. Among the two most common tasks, learned approach/aversion appeared as most frequently yielding supportive results with a little over half of studies supporting expectations. Whereas the most popular paradigm – judgment bias – had a surprisingly low number of supportive findings (40%), and yielded the only example where the authors expectation was not just not supported but actually refuted (49). Focusing on interventions, housing enrichment was by far the most studied and resulted in support of authors’ expectations 45% of the time. Studies of low birthweight had a high supportive results (75%). Supportive interventions with a single study were an electric prod (52), gestation phase (38), social hierarchy (39), injection method (51), personality (40), and serotonin depletion (36). Overall, when grouping interventions by their expected valences, cognitive tasks equally supported the author’s expectation of either positive or negative interventions.

Using a learned approach/avoidance in combination with a negatively valenced intervention was both popular and supportive of expectations compared to other combinations. However, stunning gases were only studied with this paradigm (50, 52, 54, 56). Based on human and rodent literature, exposure to stunning gases is likely to be a highly negative experience (75, 76), perhaps not requiring a particularly sensitive approach to measure differences between treatments. If other cognitive paradigms had been applied to the study of stunning gases, they might have appeared to have high supportive rates as well. Conversely, learned approach/aversion needs to be applied more often to less adverse or even positive welfare interventions to better understand its breadth of effectiveness in the detection of different welfare states.

Another popular combination was the use of judgment bias tests to assess the effect of enrichment (17, 45, 46, 48). Unfortunately, even within this combination, methodologies were heterogenous, with differences in type of task and cues used (spatial discrimination (45, 46, 48), auditory Go/No-go (17)), enrichments provided (space allowance, social partners, objects, human interaction), control conditions (space restriction, social isolation), rewards (chocolate treats, apples), and punishment used during training and tests (absence of reward, coffee bean, air puffs, toy clapper, wave of a plastic bag).

Many authors did not restrict themselves to the use of a cognitive task, and most studies included additional measures of welfare. Once again, authors displayed notable heterogeneity in their choices (e.g., serum, saliva, hair cortisol, lesions scores, vocalizations, posture, dopamine etc.). Among the 3 categories of additional welfare measures (health, physiology, and behavior), health and behavior were most frequently supportive of authors’ expectations, albeit still only about half the time, whereas physiology was well below the rate of supportive results for cognitive tasks. Furthermore, only two studies had supportive results from additional measures without supportive results from cognitive tasks.

Once again, we would like to reiterate that our ability to make overarching conclusions about the application of cognitive tasks or other measures as welfare assessment are greatly limited by the heterogeneity of the literature. For instance, the apparent validity of behavioral measures and their higher consistency with cognitive tasks is undoubtedly influenced by the highly negative interventions studied (e.g., stunning gases (5, 54), injections (21)].

Several factors beyond the heterogeneity of the literature may have contributed to the frequent lack of supportive outcomes as reflected in the failure to find significant differences between treatment groups. Challenges to the implementation of cognitive tasks include being too complex for animals to master (especially all of the animals in a group), insufficient training methods to teach the animals the task, or the tasks being not well adapted to the animals abilities and senses. Protocols usually involve extensive training, conducted in artificial conditions, and relying on potentially suboptimal cues and stimuli which were initially developed for other species. For example, much of the literature relies on visual cues, which are likely less fitting than olfactory cues in pigs (77–79). Favoring tasks that are designed around a relevance to the subjects’ ecological niches are more likely to be successful (see (35)).

A possible limitation of many paradigms is the alteration of an animal’s social environment during the test, as they are often conducted on individual animals. Social isolation has been noted to induce changes in cognitive performances in several species (80, 81). Although repeated social isolation did not affect judgment bias or cortisol levels in pigs (41), behavioral and physiological stress responses were reported when pigs were removed from their social group (82, 83). Pigs also displayed a preference for shorter term isolation compared to longer term isolation (53). Researchers are reminded to consider the effects of social isolation in their experimental designs, for example, initially training piglets as a group and gradually reducing the number of subjects until they are comfortable enough to participate alone (60) or conducting experiments where the subject can maintain visual, acoustic and olfactory contact with conspecifics.

Another potential caveat of using cognitive tasks to assess welfare is that the physical and mental engagement in the task itself might contribute to improved welfare, especially with animals raised in restrictive, unstimulating environments, as often is the case in conventional farming (84). This can be especially problematic when trying to assess the effect of environmental enrichment on performances. For example, Grimberg-Henrici et al. (58) noted that providing enriched housing to piglets slightly improved their performance in a holeboard task. However, they hypothesized that the training and testing of the task was an enrichment in itself, reducing the contrast between animals housed in enriched versus barren environments. In a study specifically looking at the effect of exposure to a cognitive task, piglets participating in a maze task early in life were suggested to have subsequent reduced fear responses and possibly lessened cognitive deficits in males (70).

Most studies explored in this review rely on food rewards as incentives for training and testing (e.g., (59, 63, 64)), but performances in cognitive tasks can be affected by anhedonic processes. Anhedonia is a depression-like condition where responsiveness to rewards such as palatable food can be decreased when an individual is in a negative affective state (85). Anhedonia has been observed in pigs, with stressed individuals who had been mixed with unfamiliar conspecifics or repeatedly restrained displaying no preference for a 0.5% sucrose solution whereas control animals did (86). Studies exploring the effects of chronic negative welfare interventions on cognitive tasks relying on food rewards should consider anhedonic processes in their interpretation.

Hunger can also impair performance in a cognitive task. In cases where a food restriction is introduced to stimulate participation in a task (e.g., (43, 63)) caution needs to be exercised as hunger has been reported to lower cognitive performances in humans (87, 88). As previously noted, an inverted U-shape relationship between hunger and cognitive performance is expected, with moderate hunger promoting engagement with the task, while high levels of hunger being detrimental to cognitive processes (89).

Authors have noted the importance of individual differences and personality traits in cognitive studies (40, 48). For instance, different sows subjected to identical housing displayed a wide range of judgment biases (from negative to positive). The aggressiveness of the animals was a better predictor of their cognitive bias than measures of physical health, such as the number of skin lesions and body condition (40). Similarly, Asher and colleagues (48) reported proactive pigs to be more optimistic in a judgment test no matter their housing enrichment, whereas reactive pigs (i.e., more passive) were more pessimistic if housed in a less enriched environment. Researchers also need to consider the possible influence of other personality traits in future studies of cognitive tasks and complement their measures with personality assessments (90, 91).

Interpretation of cognitive tasks as measures of animal welfare is further complicated by how a lack of significant differences between treatments does not necessarily reflect a failure of the experimental approach. The absence of differences in cognitive measures might reflect the failure of the hypothesized intervention to have an effect on the animal’s welfare. Due to their novelty, the sensitivity of cognitive paradigms is still under investigation. To validate the use of different cognitive tasks, efforts will first be required to determine which interventions reliably affect welfare, and whether these interventions translate to changes in cognition. Future research on cognitive tasks is encouraged, when appropriate, to consider consistency with previous work, especially for promising paradigms. Either by considering replication studies, applying previous cognitive task methodologies to test novel welfare interventions, or applying novel tasks to known welfare interventions. An exciting part of this field also is the exploration of innovative ways to include cognitive processes in the assessment of welfare, by developing novel paradigms or applying models from basic research fields. A better understanding of the potential utility of cognitive tasks for animal welfare assessment will require some continuity with, and sometimes simply replication of, existing studies. However there also remains a need for novel cognitive tasks that are used creatively to push past current boundaries in the assessment of animal welfare.

## Conclusion

This systematic review highlights the growing popularity of cognitive tasks as measures of pig welfare. However, overall rates of supportive results, i.e., changes in performance on cognitive tasks due to welfare interventions, have been limited so far, even for the most employed task, judgment bias. The numerous different combinations of experimental paradigms and welfare interventions reported in the literature creates challenges for a critical meta-analysis of the field especially in evaluating the efficiency of specific cognitive tasks in assessing animal welfare. Taken together, this review illuminates important knowledge gaps in the use of cognitive tasks that will require both further validation as well as novel innovation to ensure that their potential is fully realized in the measurement of pig welfare. Short comings in this approach to date may arise from simply not having accumulated enough similar replicates or from not yet finding the optimal cognitive task with which to measure welfare. For the field to advance, researchers need to pursue two apparently opposed research directions when applying cognitive tasks to the assessment of animal welfare: (1) Standardize and homogenize current methods to validate common and promising combinations of paradigms and welfare interventions, and (2) sustain the exploration of new improved cognitive approaches to welfare assessment.

## Data availability statement

The original contributions presented in the study are included in the article/Supplementary material, further inquiries can be directed to the corresponding author.

## Author contributions

TE and TP contributed to the conception and design of the research question. TE conducted the initial acquisition and analysis of the data, as well as the initial draft. TP revised the manuscript. All authors have approved the submitted version.

## Footnotes

^1^In our analysis of cognitive tasks and welfare interventions, the total number will exceed the number of included studies (36) due to some studies investigating multiple tasks and/or interventions.

^2^By design, studies with multiples outcomes were more likely to yield mixed outcomes
